# Retention preferences and protocols of Brazilian orthodontists: a cross-sectional study

**DOI:** 10.1590/2177-6709.29.6.e2423251.oar

**Published:** 2024-12-16

**Authors:** Daniel Gonçalves MACHADO, Daniella de Oliveira da SILVA, Júlio de Araújo GURGEL, Raphael Dutra de Resende MENDONÇA, Paula COTRIN, Karina Maria Salvatore FREITAS, Fabrício Pinelli VALARELLI, Célia Regina Maio PINZAN-VERCELINO

**Affiliations:** 1 Ceuma University, School of Dentistry (São Luís/MA, Brazil).; 2 State University of São Paulo, School of Dentistry (Marília/SP, Brazil).; 3 Ingá University Center, School of Dentistry (Maringá/PR, Brazil).

**Keywords:** Orthodontic retainers, Recurrence, Orthodontic appliances, Contenções ortodônticas, Recidiva, Aparelhos ortodônticos

## Abstract

**Objective:**

The aim of this study was to evaluate the orthodontic retention protocols used by Brazilian orthodontists.

**Methods:**

This cross-sectional study included 693 orthodontists who answered a web-based questionnaire with 22 questions regarding the participants’ characteristics, their retainers’ prescriptions, follow-up duration, and appliance fabrication.

**Results:**

A 20.84% response rate was recorded. Most orthodontists reported working in private dental offices (94.7%), and female practitioners comprised 59.9% of the respondents. The mean age of the orthodontists was 41.05 years, and the mean time practicing as an orthodontist was 11.28 years. For the maxillary arch, most orthodontists (57.9%) declared to use the Hawley retainer, and for the mandibular arch, 49.1% use the bonded retainer. Regarding the retention phase duration, 85.6% recommend using a permanent retainer for the mandibular arch; and for the maxillary arch, 36.2% recommended using it for 1 to 2 years. The main reason that led orthodontists to choose a retention design was related to the initial malocclusion characteristics (72.9%), and the most mentioned explanation for choosing the retainer type and protocol was clinical experience (44.5%). Most orthodontists (85.3%) would like to have access to general guidelines/protocols for retention procedures after orthodontic treatment.

**Conclusions:**

According to the answers, retention protocols varied among Brazilian orthodontists. Brazilian orthodontists preferred to use a Hawley retainer in the maxillary arch and a fixed straight retention bonded from canine to canine (3x3) in the mandibular arch.

## RESUMO

**Objetivo:**

O objetivo deste estudo foi avaliar os protocolos de contenção utilizados pelos ortodontistas brasileiros.

**Métodos:**

Esse estudo transversal foi composto por 693 ortodontistas que responderam a um questionário eletrônico com 22 perguntas sobre características dos respondentes, prescrição das contenções, acompanhamento das contenções e confecções dos aparelhos.

**Resultados:**

A taxa de resposta foi de 20,84%. A maioria dos ortodontistas relatou trabalhar em consultórios particulares (94,7%), e as mulheres constituíram 59,9% dos respondentes. A média de idade dos ortodontistas foi de 41,05 anos, e a média de prática como ortodontista foi de 11,28 anos. Para a arcada superior, a maioria dos ortodontistas (57,9%) relatou utilizar placa de Hawley e, para a arcada inferior, 49,1% responderam que utilizam contenção fixa. Considerando-se a duração, 85,6% recomendam o uso de contenção permanente para a arcada inferior. Para a arcada superior, 36,2% recomendam o uso por 1 a 2 anos. A principal razão para os ortodontistas definirem o tipo de contenção foi a característica inicial da má oclusão (72,9%), e a razão mais citada para o uso e protocolo de contenção foi a experiência clínica (44,5%). A maioria dos ortodontistas (85.3%) gostaria que fossem disponibilizadas diretrizes/protocolos gerais para procedimentos de contenção após o tratamento ortodôntico.

**Conclusões:**

De acordo com as respostas, o protocolo de contenção variou entre ortodontistas brasileiros, os quais relataram preferência pelo uso da placa de Hawley na arcada superior e da contenção reta fixa colada de canino a canino (3x3) na arcada inferior.

## INTRODUCTION

Despite the efforts to conduct a reasonable orthodontic treatment, predicting which treated cases will remain stable over the years is still controversial.^1^ ^-^ ^4^ Undesirable tooth movements may occur after orthodontic treatment, not only due to positional changes, but also due to age-related physiological occlusal changes.^5^ It must be considered that, in addition to the increase in life expectancy, the aesthetic demands of patients and professionals have increased,^6^ requiring satisfactory results in maintaining aesthetic and functional stability after orthodontic treatment.^5^ Therefore, the retention phase is as crucial as any other orthodontic active treatment phase, and must be carefully planned.

Several studies have evaluated the efficacy of different designs of orthodontic retainers, and the patient satisfaction with their use. However, insufficient high-quality evidence exists to establish the most effective approaches for ensuring stability in retention methods.^7^ ^,^ ^8^ The literature also describes that there seems to be no consensus among orthodontists about the actual need, design, or recommendations concerning the length of time the orthodontic retainers should be used.^9^ ^-^ ^20^ Moreover, differences among countries may occur, suggesting the need for further studies in different regions.^9^ ^-^ ^20^

Preexisting studies on trends and retention protocols showed the preference of North American, European, and Oceania orthodontists.^9^ ^-^ ^19^ For the maxillary arch, Hawley plate was the most commonly prescribed retainer in the United States,^16^ ^,^ ^18^ and Lithuania;^11^ the vacuum-formed retainer, in Canada,^12^ Australia,^13^ New Zealand^14^ and United Kingdom;^19^ the combination of maxillary fixed and removable retainers, in Norway^9^ and Netherlands;^15^ and in Switzerland^10^, bonded retainer was the most reported design. Mandibular fixed retainers are used by most orthodontists worldwide.^9^ ^,^ ^10^ ^,^ ^12^ ^-^ ^18^ In Lithuania,^11^ a combination of a bonded and a removable retainer was mostly cited by the orthodontists for the mandibular arch; and in the United Kingdom,^19^ their preference was for the use of vacuum-formed retainers. In terms of the duration of wear, indefinite retention was reported by more than 82% of the respondents in Switzerland,^10^ Canada,^12^ New Zealand,^14^ Netherlands,^15^ ^,^ ^17^ and the United States.^16^ ^,^ ^18^ However, there is a lack of studies evaluating orthodontists from Latin America. The authors of the present study did not find studies evaluating the retention protocols used by Brazilian professionals. Thus, this study aimed to evaluate the preferred retention protocol used by Brazilian orthodontists. 

## MATERIAL AND METHODS

This cross-sectional study received approval from the human research ethics committee of the Centro Universitário do Maranhão/Brazil (protocol: 3.748.016), and an informed consent was obtained from all participants.

This survey was carried out with Brazilian orthodontists registered as specialists in Orthodontics at the Brazilian Association of Orthodontics (ABOR). The inclusion criterion for the specialists was having concluded the graduate orthodontic course.

According to data from the Federal Council of Dentistry website, when the study began (2020), there were 27,280 orthodontists in Brazil (15,984 women and 11,296 men). The sample calculation was performed using this data, considering a confidence level of 4% and a test power of 95%. A minimum sample of 588 orthodontists was required.

An online questionnaire (Google Forms) was used, generating a database with the responses. The questionnaire was available for responses from July to August 2020. In the questionnaire’s introduction, the informed consent approved by the human research ethics committee was described, and the subjects were informed about the study objectives. The questionnaire was anonymous, and no personal identification was required. A link to the questionnaire was randomly emailed to 3,324 orthodontists registered as members of the Brazilian Association of Orthodontics. The e-mails were randomly selected by drawing from the five Brazilian regions.

The questionnaire was based on and adapted from previously published research.^9^ ^,^ ^11^ ^,^ ^14^ ^-^ ^17^ The questionnaire contained 22 questions oriented in the following sections: data regarding participant characteristics (sex, age, region of orthodontic practice, area of orthodontic education); retainers’ prescriptions and retention follow-up (Questionnaire 1). 

**Questionnaire 1:** Web-based questionnaire with 22 questions regarding the participants’ characteristics, retainer prescription, retention follow-up, and appliance fabrication.

Questionnaire
1- How old are you? ________________
2- Sex:
( ) Male
( ) Female
3- What is the region of your main place of orthodontic practice?
( ) Southeast
( ) South
( ) Midwest
( ) Northeast
( ) North
4- Where did you acquire your specialist orthodontic qualification?
( ) Southeast
( ) South
( ) Midwest
( ) Northeast
( ) North
( ) other: specify_______
5- How long ago did you acquire your specialist orthodontic qualification? _____
6- How many days per week do you work with orthodontic patients? _________
7- What clinical setting best describes your practice?
( ) Private
( ) Community/hospital/university
8- What retainer do you MOST COMMONLY use in the MAXILLARY arch?
( ) Hawley type of retainer (or similar)
( ) Vacuum-formed retainer
( ) Fixed retainer bonded to incisors
( ) Fixed retainer bonded to all six anterior teeth (canine-canine)
( ) Both a fixed retainer and a removable appliance are used simultaneously
( ) Other
9- What retainer do you MOST COMMONLY use in the MANDIBULAR arch?
( ) Hawley type of retainer (or similar)
( ) Vacuum-formed retainer
( ) Fixed retainer bonded to all six anterior teeth (canine-canine)
( ) Fixed retainer bonded to canines only
( ) Fixed hygienic retainer
( ) Other
10- How long do you recommend wearing the MAXILLARY arch retainer?
( ) 0 - 6 months
( ) 6 months - 1 year
( ) 1 - 2 years
( ) 2 - 3 years
( ) 3 - 5 years
( ) Permanently
( ) Other
11- How long do you recommend wearing the MANDIBULAR retainer?
( ) 0 - 6 months
( ) 6 months - 1 year
( ) 1 - 2 years
( ) 2 - 3 years
( ) 3 - 5 years
( ) Permanently
( ) Other
12- What reasons influence your choice of a specific type of retainer? (Use several options if applicable)
( ) Initial malocclusion
( ) Final result
( ) Oral hygiene
( ) Periodontal health
( ) Patient’s motivation and desire
( ) Patient’s age
( ) Myofunctional condition
( ) Tooth morphology
( ) Third molars
13- What is the most important reason for using your retention protocol?
( ) Orthodontic education
( ) Clinical experience
( ) Scientific literature
( ) Courses
( ) Discussion with colleagues
14- Have you changed your retention protocol in the last 5 years?
( ) Yes
( ) No
15- What kind of retention information do you give your patients? (Use several choices if applicable)
( ) Verbal
( ) Written
( ) Information about precautions and possible problems (loss, retainer detachment, and/or breakage)
( ) Use of interdental brush
( ) Use of dental floss
16- How many retention appointments are scheduled in the FIRST YEAR AFTER orthodontic active treatment?
( ) 0
( ) 1
( ) 2
( ) 3
( ) 4 or more
17- How many retention appointments PER YEAR are scheduled AFTER THE FIRST YEAR of retention?
( ) 0
( ) 1
( ) 2
( ) 3
( ) 4 or more than 4 times
18- If retention is scheduled for over 3 years, who does the follow-up?
( ) Myself
( ) Patient’s general dentist
( ) Other
19- Who fabricates the REMOVABLE retainers used in your clinic?
( ) Made by myself
( ) Made by the assistants
( ) Made at a commercial lab
( ) Made at a specialized company (for example, Invisalign)
( ) Other
20- Who fabricates and installs the FIXED retainers used in your clinic?
( ) Made and bonded by myself
( ) Made by myself, but bonded by an assistant
( ) Made and bonded by an assistant
( ) Made by an assistant, but bonded by myself
( ) Made at a commercial lab, but bonded by myself
( ) Made at a commercial lab, but bonded by an assistant
21- What type of wire do you most commonly use for fixed retainer fabrication?
( ) Single-strand round wire
( ) Single-strand rectangular wires
( ) Braided wire
( ) Other
22- Would it be advantageous to have general guidelines for retention procedures after comprehensive orthodontic treatment?
( ) Yes
( ) No
( ) Uncertain

The first questions about retainer prescriptions comprised design, duration, reasons to choose the appliance, the preferred protocol and its alterations in the last 5 years, and instructions about the retainers and their maintenance. In order to evaluate retention management, orthodontists were asked about the appointment schedules after the retainers’ installation. The next set of questions concerned the appliance’s fabrication. Finally, the orthodontists could express their opinions on the need for a clinical practice guideline for retention after active orthodontic treatment.

The questionnaire was initially tested on a pilot population before its administration in this study. The pilot study was undertaken with 19 orthodontists, to evaluate the clarity of the questions, and the language used. Some questions were rewritten, changing some words by synonyms, so that the orthodontists would be more likely to understand. The participants of the pilot study were not included in the main study.

Descriptive statistics were performed with percentages for each question/answer. Statistical analysis was performed with SPSS software (version 20, IBM, Armonk, NY, USA). 

## RESULTS

A total of 693 orthodontists answered the survey, giving a response rate of 20.84%. Female orthodontists comprised 415 (59.9%) of the respondents. The mean age of the orthodontists was 41.05 ± 10.50 years. According to the answers provided, the average time of experience as an orthodontist was 11.28 ± 9.11 years.

Most orthodontists reported working in the Southeast region of Brazil (280, 40.4%). Forty-five percent reported working 5 days a week. The average weekly days that the orthodontists work in the dental office was 4.24 ± 1.52 days. Most of them (94.7%) declared working in private dental offices.

When asked about where they acquired their specialist orthodontic qualification, 352 (50.8%) reported having acquired their graduation in the Southeast region of Brazil.

Most orthodontists declared using the Hawley retainer for the maxillary arch (57.9%) and fixed canine-to-canine retainer bonded in incisors (49.1%) for the mandibular arch (Table 1).

**Table 1:** Summary of questionnaire responses regarding the type of retainer and retention wear protocol.

TYPE OF RETAINER	%
Maxillary arch	
Hawley retainer	57.9
Vacuum-formed retainer	19.2
Bonded 12-22 (bonded to 4 teeth)	5.2
Bonded 13-23 (bonded to 6 teeth)	4.8
Bonded and removable retainers are used simultaneously	11
Other	1.9
Mandibular arch	
Hawley retainer	1.6
Vacuum-formed retainer	4.2
Bonded 33-43 (bonded to 6 teeth)	49.1
Bonded 33-43 (bonded to canines only)	13.4
Fixed hygienic retainer	29.7
Other	2
RETENTION WEAR PROTOCOL	%
Maxillary arch	
0 - 6 months	0.5
6 months - 1 year	14.7
1 year - 2 years	36.2
2 years - 3 years	11.8
3 years - 5 years	5.2
Permanently (for life)	23.6
Other	8
Mandibular arch	
0 - 6 months	0.5
6 months - 1 year	0.3
1 year - 2 years	2.3
2 years - 3 years	2.6
3 years - 5 years	4.3
Permanently (for life)	85.6
Other	4.4

Most orthodontists reported prescribing a shorter duration of retainer wear for the maxillary than for the mandibular arch (Table 1). The orthodontists more often recommended a period of 1 to 2 years for the use of the retainer in the maxillary arch (36.2%) and permanent (for life) in the mandibular arch (85.6%). 

Table 2 describes the reasons that lead orthodontists to choose a type of retention: Initial malocclusion (72.9%), oral hygiene (59.4%), periodontal health (54.8%), final results (51.7%), compliance and choice of the patient (36.6%), age of the patient (24.5%), myofunctional condition (36.6%), dental morphology (14.7%) and third molars (3.5%) were indicated. 

**Table 2:** Percentage of orthodontists indicating the reasons for choosing the type of retention.

Reasons	%
Initial malocclusion	72.9
Final result	51.7
Oral hygiene	59.4
Periodontal health	54.8
Patient’s motivation and desire	36.6
Patient’s age	24.5
Myofunctional condition	36.6
Tooth morphology	14.7
Third molars	3.5

The reasons for the use of the retainer type and protocol mentioned by the orthodontists were: clinical experience (44.5%), having learned in orthodontic education (29%), scientific literature (18.3%), learned in other orthodontic courses (5%), and after discussion with colleagues (3.2%). The same retention protocol was used for more than 5 years by 51.6% of the specialists. All of the orthodontists declared giving instructions for the use of retainers to their patients. Data showed more verbal than written instructions; and great concern regarding precautions and possible problems, such as retainer breakage/loss, and oral hygiene, was reported (Table 3). 

**Table 3:** Percentage of information concerning retention given by the orthodontists.

Information	%
Verbal	89.6
Written	35.2
Information about precautions and possible problems (loss, retainer detachment, and/or breakage)	81.7
Use of interdental brush	40.6
Use of dental floss	80.5

In the first year after the conclusion of active treatment, most orthodontists (43.8%) reported scheduling three follow-up appointments. After the first year, most orthodontists (45.4%) reported scheduling two dental appointments (Table 4). When the retention was defined to be used for more than three years, most orthodontists (91.9%) reported scheduling a follow-up appointment (Table 4).

**Table 4:** Summary of questionnaire responses regarding retention management and retainer manufacture.

RETENTION MANAGEMENT	
How many retention appointments are scheduled in the FIRST YEAR AFTER orthodontic treatment?	%
None	2.2
1	4.6
2	31.1
3	43.8
4 or more	18.3
How many retention appointments per year are scheduled AFTER THE FIRST YEAR of retention?	%
None	5.8
1	26.4
2	45.4
3	11.4
4 or more	11
If retention is scheduled for longer than 3 years, who does the follow-up?	%
Myself	91.9
Patient’s general dentist	6.1
Other	2
RETAINER MANUFACTURE	
Who fabricates the REMOVABLE retainers used in your clinic?	%
Made by myself	13.7
Made by the assistants	5.5
Made at a commercial lab	76.2
Made at specialized company (for example, Invisalign)	4
Other	0.6
Who fabricates and installs the FIXED retainers used in your clinic?	%
Made and bonded by myself	53.2
Made by myself, but bonded by an assistant	1
Made and bonded by an assistant	0.6
Made by an assistant, but bonded by myself	1.8
Made at a commercial lab, but bonded myself	42.1
Made at a commercial lab, but bonded by an assistant	1.3
What type of wire do you most commonly use for fixed retainer fabrication?	%
Single-strand round wire	67.4
Single-strand rectangular wires	2.4
Braided wire	26.4
Other	3.8

According to the responses, most removable retainers were manufactured by laboratory prosthetics (76.2%), and most bonded retainers were fabricated and bonded by the orthodontists themselves (53.2%) (Table 4). For the bonded retainers, 67.4% declared using single-strand round wires, braided wires (26.4%), and single-strand rectangular wires (2.4%) (Table 4).

Most orthodontists (85.3%) think it would be advantageous to have general guidelines/protocols for retention procedures after orthodontic treatment; 9.1% did not, and 5.6% were uncertain.

## DISCUSSION

The average time of experience as an orthodontist was 11.28 years. A similar study conducted in Lithuania^11^ found a similar period of orthodontics practice. However, the experience time observed in the present study was lower than Switzerland,^10^ Netherlands,^15^ Australia,^13^ and Canada.^12^ Most orthodontists work in private dental offices, which agrees with the studies from Lithuania^11^ and Australia.^13^

The Hawley retainer was the preferred retainer for the maxillary arch by most Brazilian orthodontists (57.9%), corroborating with the findings in the United States,^16^ ^,^ ^18^ and Lithuania.^11^ In the United Kingdom,^19^ New Zealand,^14^ Australia,^13^ and Canada,^12^ they prefer the vacuum-formed retainers; while in Norway^9^ and Netherlands,^15^ most orthodontists use a combination of fixed and removable maxillary retainers. These differences point to the lack of consensus regarding the preference for maxillary retainers. 

The fixed straight canine-to-canine mandibular retainer bonded in all incisors was preferred by 49.1% of Brazilian orthodontists. This is in accordance with the findings obtained in the United States,^16^ ^,^ ^18^ Norway,^9^ Netherlands,^15^ ^,^ ^17^ New Zealand,^14^ Australia,^13^ Switzerland,^10^ and Canada.^12^ The deterioration of alignment in the mandibular anterior teeth is one of the most notable characteristics in the patient’s physiological maturation process.^5^ Moreover, it is crucial to highlight that contemporary patients have higher expectations, and even minor irregularities are no longer tolerated.^15^ Therefore, the recommendation of a fixed retainer may ensure that the alignment achieved at the end of orthodontic treatment will be maintained for many years.^21^ ^-^ ^23^ Furthermore, achieving adequate compliance for the use of removable retainers can be challenging.^24^

Most orthodontists in Brazil recommended using maxillary orthodontic retainers for 1 to 2 years post-treatment. There seems to be no consensus on this subject.^25^ Therefore, one could speculate that the preference for this period of use is due to the relapse that occurs in the alignment immediately after debonding.^26^

Most Brazilian orthodontists recommend the use of mandibular fixed retainers for life. This recommendation is followed by orthodontists from the Netherlands,^15^ ^,^ ^17^ United States,^16^ Norway,^9^ Australia,^13^ and Canada.^12^ These results are probably related to the findings in the literature that showed the long-term changes in the alignment over the years.^1^ ^-^ ^5^

The primary factor influencing the choice of the retention protocol was the clinical experience. Possibly due to the absence of a standardized protocol or a consensus on the most suitable orthodontic retention protocol, Brazilian orthodontists tend to mostly base their choices on their clinical experiences, rather than relying heavily on scientific literature. Although there is no ideal retention protocol, orthodontists must base their clinical decisions on the available scientific evidence, regarding the type, regimen, survival, cost-effectiveness, and patient’s compliance with retainers use.^25^ ^,^ ^27^

The main reasons that led Brazilian orthodontists to choose the retainer design were initial malocclusion, oral hygiene, periodontal health, and final treatment results (51.7%). In previous studies, the pretreatment situation was also considered by most of the orthodontists as important in determining retainer choices.^11^ ^-^ ^13^ ^,^ ^17^

All orthodontists reported giving instructions about the retainer to the patients. The guidelines on orthodontic retention are mostly given verbally. This information relies on precautions and problems that can occur during this phase, focusing on retainer loss, detachment and/or breakage, and oral hygiene. These data suggest a greater concern with periodontal health when using orthodontic retainers.

During the first year of follow-up, there was a preference for three checkup appointments (43.8%), in accordance with previous findings.^11^ ^,^ ^15^ This finding is essential since, when retention checkups are performed, the patient’s compliance can be positively influenced.^28^ Despite the importance of the retention phase, one percent of the professionals did not see their patients subsequently, corroborating with the findings of Renkema et al.,^17^ which emphasized that some professionals gave the responsibility for the retention phase entirely to the patient and/or the general dentist. After the first year, dentists recommend two annual checkups, and the orthodontist or another dentist could do this control. This result also emphasizes the concern to ensure good oral hygiene in the retention phase.

The most frequently chosen material to fabricate the fixed orthodontic retention was single-strand round wires, corroborating with the findings in Australia^13^ and Canada.^12^ It can be speculated that the single-strand wire was preferred due to the reduced plaque accumulation around it.^29^ Single-strand steel wires are also widely available in the orthodontic market. However, a recent systematic review showed no evidence of difference between retainers regarding periodontal health.^30^

Most orthodontists feel that having general guidelines/protocols for retention procedures after orthodontic treatment would be beneficial, in agreement with previous studies.^11^ ^,^ ^17^ The mixed responses in this survey demonstrated the need to develop an evidence-based practice guideline for procedures after orthodontic treatment. This finding is in accordance with Martin et al.,^30^ who claimed that it is currently impossible to draw firm conclusions about any approach to retention over another. More high‐quality studies are needed to measure tooth stability over the years, how long retainers last, patient satisfaction, and adverse side effects from wearing retainers, such as tooth decay and gingival disease. 

Although there is no validated questionnaire in Brazil regarding the retainer protocol, the questionnaire used was similar to those applied in previous studies,^9^ ^,^ ^11^ ^,^ ^14^ ^-^ ^17^ facilitating a closer comparison with the results. Moreover, a pilot questionnaire was applied to Brazilian specialists before the study began, to ensure its validity and acceptability. It is essential to highlight that online questionnaires allow easier access and preserve participants’ anonymity, reducing the tendency of socially desirable responses. Nevertheless, the methodology expanded the sample to all Brazilian regions and covered different age ranges. Additional questions could provide more information; however, they could decrease the number of participants, since they could take more time to be answered.^11^ Further studies are recommended to expand the knowledge in this subject.

## CONCLUSIONS

Most Brazilian orthodontists prefer a Hawley retainer for the maxillary arch and a bonded retainer for the mandibular arch. The choice of the retention protocol is mainly based on clinical experience, as well as on pretreatment characteristics of the malocclusion. Most orthodontists would like general guidelines/protocols for retaining procedures after orthodontic treatment.
